# Agreement in extreme precipitation exposure assessment is modified by race and social vulnerability

**DOI:** 10.3389/fepid.2023.1128501

**Published:** 2023-03-02

**Authors:** Kyle T. Aune, Benjamin F. Zaitchik, Frank C. Curriero, Meghan F. Davis, Genee S. Smith

**Affiliations:** ^1^ Johns Hopkins Bloomberg School of Public Health, Department of Environmental Health and Engineering, Johns Hopkins University, Baltimore, MD, United States; ^2^ Johns Hopkins Krieger School of Arts and Sciences, Department of Earth and Planetary Sciences, Johns Hopkins University, Baltimore, MD, United States; ^3^ Johns Hopkins Bloomberg School of Public Health, Department of Epidemiology, Johns Hopkins University, Baltimore, MD, United States; ^4^ Johns Hopkins Medicine, Department of Molecular and Comparative Pathobiology, Johns Hopkins University, Baltimore, MD, United States; ^5^ Hopkins Center for Health Disparities Solutions, Johns Hopkins Bloomberg School of Public Health, Baltimore, MD, United States

**Keywords:** climate change, climate justice, remote sensing, spatial analysis, gridded climate data, extreme precipitation events, race

## Abstract

Epidemiologic investigations of extreme precipitation events (EPEs) often rely on observations from the nearest weather station to represent individuals' exposures, and due to structural factors that determine the siting of weather stations, levels of measurement error and misclassification bias may differ by race, class, and other measures of social vulnerability. Gridded climate datasets provide higher spatial resolution that may improve measurement error and misclassification bias. However, similarities in the ability to identify EPEs among these types of datasets have not been explored. In this study, we characterize the overall and temporal patterns of agreement among three commonly used meteorological data sources in their identification of EPEs in all census tracts and counties in the conterminous United States over the 1991–2020 U.S. Climate Normals period and evaluate the association between sociodemographic characteristics with agreement in EPE identification. Daily precipitation measurements from weather stations in the Global Historical Climatology Network (GHCN) and gridded precipitation estimates from the Parameter-elevation Relationships on Independent Slopes Model (PRISM) and the North American Land Data Assimilation System (NLDAS) were compared in their ability to identify EPEs defined as the top 1% of precipitation events or daily precipitation >1 inch. Agreement among these datasets is fair to moderate from 1991 to 2020. There are spatial and temporal differences in the levels of agreement between ground stations and gridded climate datasets in their detection of EPEs in the United States from 1991 to 2020. Spatial variation in agreement is most strongly related to a location's proximity to the nearest ground station, with areas furthest from a ground station demonstrating the lowest levels of agreement. These areas have lower socioeconomic status, a higher proportion of Native American population, and higher social vulnerability index scores. The addition of ground stations in these areas may increase agreement, and future studies intending to use these or similar data sources should be aware of the limitations, biases, and potential for differential misclassification of exposure to EPEs. Most importantly, vulnerable populations should be engaged to determine their priorities for enhanced surveillance of climate-based threats so that community-identified needs are met by any future improvements in data quality.

## Introduction

As the frequency and intensity of extreme precipitation events (EPEs) increases due to warming atmospheric temperatures and land use changes, interest in epidemiologic investigations of the negative health effects of EPEs have increased as well. EPEs have previously been found to increase the risk of injury and drowning (1–3), enteric illness (1, 3–6), vector-borne infectious diseases (7–9), and allergic diseases (10–12), among others (13–15). However, many of these previous studies rely on assignment of precipitation measured at a single weather station's location to represent the average exposure experienced by a study's population of interest. Sometimes these exposures are assigned to relatively small geographic areas, but other times a single station's observations are meant to represent an area as large as a county (16–19). As many quality-controlled weather stations are located at airports outside of population centers, it is possible, perhaps even likely, that the meteorological conditions experienced by a study population could differ systematically and substantially from the conditions measured and assigned to represent their exposure to extreme weather events like EPEs. This may be particularly true in rural areas where official weather stations are spatially scarce. Environmental and community determinants of health also vary across space, raising the possibility that the likelihood of exposure misclassification bias related to weather station location may vary differentially across classes of sociodemographic vulnerabilities relevant to the health impacts of EPEs.

A proposed improvement to these methodological limitations was the use of high spatial resolution gridded climate datasets that involve spatial interpolation or statistical downscaling of weather stations observations, sometimes incorporating remote sensing data as well, to produce spatially resolved estimates of meteorological conditions across an entire geography. In studies of the health impacts of extreme heat events, researchers have found that the use of such datasets, and the choice between gridded climate products, can significantly impact the measures of association (20–24), suggesting that the exposure data source decisions should be evaluated carefully and with a full understanding of candidate data sources' strengths, limitations, and biases. While the mechanisms responsible for fine spatial variability in temperature related to factors like land use, artificial and natural albedo, proximity to water bodies, and urban heat island effect differ from the mechanisms driving spatial patterns in precipitation and EPEs, regional and local feedback systems related to land use, atmospheric aerosol concentrations, and temperature can strongly influence the patterns of EPEs at small spatial scales (25–29). However, the authors are unaware of similar investigations into the various effects that data source selection may have on future epidemiologic investigations into the health effects of EPEs or other precipitation-related meteorological threats to health. Furthermore, while meteorological validation studies have identified systematic geographic and orographic biases in climate datasets, biases and errors have not been evaluated along sociodemographic, racial, and economic lines. Beyond scientific interest in the role that differential exposure misclassification might have on study findings, there may be serious climate and environmental justice implications for communities for whom accurate measurements of exposures to dangerous climate hazards are not available.

Therefore, the objectives of this study were to characterize the overall and temporal patterns of agreement among three commonly used, but variably rigorous, meteorological data sources and methods in their identification of EPEs in the conterminous United States (CONUS) from 1991 to 2020 and to evaluate the association between sociodemographic population characteristics with agreement in EPE identification. We considered three common data sources, (1) weather stations from the U.S. Global Historical Climatology Network (GHCN), (2) interpolated gridded climate data from the Parameter-elevation Relationships on Independent Slopes Model (PRISM), and (3) the model and observation informed meteorological fields of the North American Land Data Assimilation System (NLDAS). We compared agreement in identification of EPEs among these three data sources across commonly identified vulnerability factors and using the Centers for Disease Control and Prevention's social vulnerability index (SVI), and finally explored the practical effects of differences in agreement at three hypothetical example study locations in rural Pennsylvania, a Native American Reservation in Washington, and New Orleans, Louisiana.

## Materials and methods

### Geographic data sources and methods

Many epidemiologic investigations into the effects of climate and extreme weather on human health compare meteorological data with geospatial health data aggregated to some administrative spatial unit. To analyze and provide useful summaries for future epidemiologic investigations into extreme weather and climate events, two levels of geographic units were considered as exposure assessment locations for the present analysis, census tract and county borders, two of the most commonly used areal units to which health data are aggregated in the United States. Shapefiles for census tracts (*N* = 72,333) and county boundaries excluding water area (*N* = 3,108) were downloaded from the U.S. Census Bureau's TIGER/Line database using the “tigris” package in R (30). The most recent 2019 shapefiles were used, and all census data sources were filtered for only those states and the District of Columbia within the CONUS to allow for direct comparison among meteorological data sources, some of which are limited to that geographic region. Eight census tracts (coastal islands and nature/aquatic reserves) in the CONUS were located outside the coverage area of one or more of the meteorological data sources and were therefore excluded from analysis, leaving 72,325 census tract exposure assessment locations.

### Meteorological data sources

Meteorological data were obtained from a variety of sources from 1991 to 2020, the same period of time described by the recently released U.S. Climate Normals (31). To evaluate exposure assessments of EPEs, three classes of data sources, selected in increasing degree of complexity, were considered. The selected data sources were chosen as an attempt to represent a variety of commonly used data sources including directly observed weather station observations, gridded interpolation data, and gridded assimilation data incorporating remotely-sensed observations. While the data sources included here represent a non-exhaustive list of those available and used in epidemiologic research, the selected datasets aim to demonstrate limitations in exposure assessment so that future research using these three common classes of data might be better guided toward more accurate analyses. First-order data sources, that is, raw, unadjusted observations at weather stations throughout the CONUS were taken from the National Oceanic and Atmospheric Administration's GHCN (32). This dataset represents a subset of all available weather observations within its Cooperative Observer Program that undergo rigorous quality assurance and quality control processes and thus represent some of the highest accuracy first-order weather observations available in the world. GHCN daily observations are available from the year 1833 to present and record information on five core elements – precipitation, snowfall, snowfall depth, maximum temperature, and minimum temperature – with numerous other supplementary elements. Although rigorous quality control and assurance practices are applied to these data, these policies do not provide for consistent data collection and therefore spatial and temporal gaps in data availability are present.

Beyond first-order, point-level observations, many gridded climate datasets exist that might be used by epidemiologists to estimate meteorological conditions at any location in the CONUS. These gridded datasets are constructed with varying numbers of contributing data sources and factors and with variable complexity. The first gridded data source considered in the present investigation was PRISM (33, 34). PRISM is a spatial interpolation model that uses observations from many U.S. weather networks, including GHCN and the Cooperative Observer Program, and applies spatial weighting to account for differences in precipitation across elevation and orography to estimate values on a 4 km × 4 km grid across the CONUS. PRISM data are available from the year 1981 to present and estimate daily precipitation, minimum temperature, mean temperature, maximum temperature, dew point, minimum vapor pressure deficit, and maximum vapor pressure deficit.

A second, more complex gridded dataset frequently used in climate and epidemiologic literature is the Land Data Assimilation System developed and maintained by the National Aeronautics and Space Administration (NASA). Specifically, the model developed specifically for North America, NLDAS, was selected for the present investigation (35, 36). This data source contains estimates created by combining a regional atmospheric model with surface station observations, spatial interpolation modeling, and satellite observations from the Geostationary Operational Environmental Satellites and low-earth Polar Orbiting Environmental Satellites. Hourly NLDAS data are available from the year 1979 to present over a 0.125° × 0.125° (∼12.5 km × 11.5 km at CONUS latitudes) grid across the CONUS and estimate precipitation, temperature, specific humidity, wind speed and direction, surface pressure, and downward radiation.

### Extreme weather event definitions

Broadly speaking, EPEs were considered due to the severity of their effects on population health, the increased frequency with which they are predicted to occur in the coming decades due to climate change, and the high confidence with which those predictions are made (14, 28, 37). There are multiple possible mechanisms by which EPEs might negatively affect health (13), some of which may overwhelm wastewater and stormwater handling infrastructure with more precipitation than is typical for a given location, and others that influence behavioral risk factors through heavy precipitation beyond a fixed threshold, regardless of what is locally typical.

To replicate commonly used methods and provide useful recommendations to future epidemiologic studies on climate and health, two definitions of EPEs were used – a locally specific and a more general threshold for extreme events were both included in the present investigation. To provide locally specific thresholds, distributions of daily observed precipitation values on days with precipitation were drawn for each location and in keeping with previously published methods (38), EPEs were defined as days where the observed daily precipitation exceeded the 99th percentile of total days with precipitation from 1991 to 2020. While absolute thresholds vary in the published literature (39), a more general threshold for EPEs was also considered and defined here as daily precipitation of more than one inch (2.54 cm), consistent with previous investigation (40).

### First-Order meteorological data processing

Precipitation data (in tenths of millimeters) from 35,334 GHCN stations within the CONUS in operation from 1991 to 2020 were identified and all observations were downloaded using the “rnoaa” package (41) resulting in 131,765,655 total observations. All observations with quality issues flagged by NOAA were removed from the analysis (N = 6,235, 0.005%). All non-flagged observations were considered valid and therefore included in the analysis (*N* = 131,759,420) after being converted into millimeters for consistency with the remaining data.

**Figure 1:**
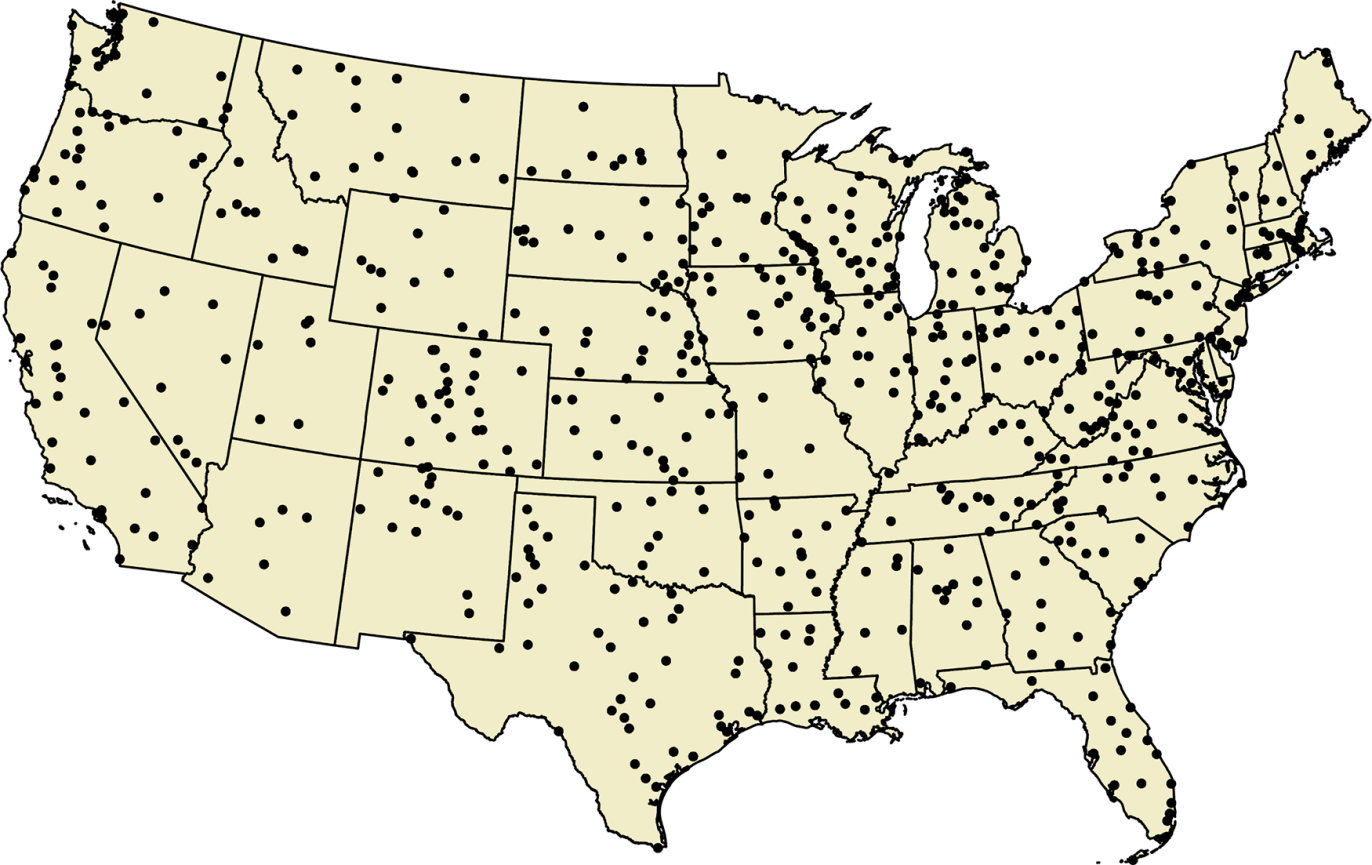
GHCN stations used in EPE threshold calculation, 1991–2020. Locations of GHCN stations with ≥99% complete daily observations in the CONUS from 1999 to 2020 included in EPE threshold calculation.

To create the 99th percentile thresholds for EPEs, stations were subsetted if they had 99% complete daily precipitation observations, consistent with previously published methods (42). This strict inclusion criteria for the distribution-based thresholds ensures that the threshold for each included station is entirely representative of the climate of that location. Of all the GHCN stations in operation from 1991 to 2020, 648 stations were included in the EPE threshold calculation subset and these included stations provide good geographic coverage across the CONUS (Figure 1). Pairwise distances between the locations of these subsetted stations with 99% complete observations and the exposure assessment locations (centroids of census tracts and counties) were calculated and the locally specific EPE thresholds of each tract and county were assigned as the threshold of the nearest included station.

Next, daily precipitation measurements were assigned to each exposure assessment location. Matrices of pairwise distances between all station locations and exposure assessment locations were created and the value of the closest station in operation on each day was assigned to each exposure assessment location. Since strict inclusion criteria for complete observations were not applied here, these were often different stations than those from which local 99th percentile thresholds were calculated. However, thanks to rigorous quality control in the underlying GHCN data, these values still represent accurate daily measurements. While averaging of nearby stations or employing inverse distance weighting could produce a potentially more representative estimate of experienced precipitation, only the nearest station's value was considered to represent the simplest methods that many environmental exposure assessments employ. Daily EPE occurrences were then calculated for each exposure assessment location as days with assigned precipitation values exceeding the defined EPE thresholds, the local 99th percentile and one inch.

### Gridded climate data processing

Daily PRISM data for total precipitation (in millimeters) were downloaded from the PRISM FTP site for 1991–2020 (43) as geoTIFF rasters (*N* = 10,958 daily files) with 4 × 4 km spatial resolution (*N* = 9,560,909,790).

Hourly NLDAS data for total precipitation (in kg/m^2^, equivalent to millimeters) were downloaded from the NASA Goddard Earth Sciences Data and Information Services Center (44) for 1991–2020 as GRIB raster bricks (*N* = 262,992 hourly files) using wget. These hourly rasters were summarized to daily values by calculating the raster pixel sum of hourly precipitation totals for each day (*N* = 10,958 daily files) at 0.125° × 0.125° spatial resolution (*N* = 1,294,095,968).

Daily values for PRISM and NLDAS data at census tracts and counties were assigned as the spatially weighted average of all cell values contained within or intersecting a polygon's border. EPE thresholds were created for each exposure assessment location as the 99th percentile of daily values assigned to that location. Finally, daily EPE occurrences were identified for each location as described previously.

### Statistical analyses

Agreement in identification of each definition of EPEs (as binary variables: 1, EPE; 0, No EPE) was assessed by calculating Cohen's kappa statistic for each two-way comparison of data sources – GHCN vs. PRISM, GHCN vs. NLDAS, and PRISM vs. NLDAS. Cohen's kappa (45) is a measurement of percent agreement between two raters in categorizing an observation as a discrete outcome that takes into account the likelihood of chance agreement and ranges from 0 to 1. Landis (46) provide guidance for the practical interpretation of kappa statistics with scores < 0.00 considered as poor, 0.00–0.20 as slight, 0.21–0.40 as fair, 0.41–0.60 as moderate, 0.61–0.80 as substantial, and 0.81–1.00 as almost perfect. Modification of agreement in EPE identification among the data sources was explored across strata of variables related to time, space, and population characteristics. Since each data source has undergone changes during the time period of interest, longitudinal temporal differences in agreement were explored through calculation of annual kappa statistics.

Because of substantial time-varying differences in agreement over the entire 1991–2020 period, the 2011–2020 time period was used to explore the effect of location characteristics on EPE identification agreement from using data from the U.S. Census Bureau using the R package “tidycensus” (47). Evaluation of this time periods limits the contribution of systematic changes to the datasets from our evaluation of their performance across social vulnerability factors. Because counties are geographically large, do not exhibit uniform distribution of underlying population characteristics, and often exhibit more variability within a given county than among a group of counties, these analyses were performed only at the census tract level. Population demographic differences were explored through calculation of a kappa statistic for strata of locations according to % minority population, % Black population, % Native American population, % Asian population, % Hispanic population, and % population in a household below the federal poverty threshold using the average of the 2011–2015 5-year American Community Survey (ACS) estimates and the 2016–2020 ACS estimates. Tracts were categorized as majority Black, majority Native American, majority Asian, majority Hispanic, or majority minority (i.e., < 50% non-Hispanic White) using dichotomous indicator variables such that each tract was categorized as majority Black or not majority Black, and so on for each racial distinction. Tracts were classified as low income or not low income according to the U.S. Treasury Department's definition as tracts with >20% of residents below the federal poverty threshold (48). Tracts were also classified according to U.S. Census Bureau urbanicity designation as either rural, urban cluster, or urban. Finally, a measure of overall social vulnerability of tracts was considered using the U.S. Centers for Disease Control and Prevention Agency for Toxic Substance and Disease Registry's SVI composite index for overall vulnerability from 2018 (49). Briefly, SVI scores represent a percentile ranking of all U.S. tracts' values in four domains – socioeconomic, household composition and disability, minority status and language, and housing type and transportation – with higher scores representing higher social vulnerability. For comparisons, SVI was categorized as very low (0.00–0.25), low (0.25–0.50), high (0.50–0.75), and very high (0.75–1.00).

Given the interdependencies of the data sources (PRISM interpolates GHCN station data; NLDAS incorporates the interpolated GHCN data from PRISM) and the fact that the primary determining factor behind measurement uncertainty in these models is distance from the GHCN input data, the effect of the distance from a census tract to the nearest GHCN station was investigated. Since GHCN station availability is not constant over time, the distance from the nearest station to the centroid of each census tract was calculated for each day from 2011 to 2020 and the mean of these daily distances for each census tract was considered for further analyses. Daly (33), in detailing the methods for PRISM interpolation, describe statistical clustering of observation values between GHCN stations within 7–10 km of one another and subsequently downweight the values of stations <7 km from one another, so census tracts with 7 km of the nearest GHCN station were considered as a stratum of locations for which agreement among the data sources would be expected to be highest. The radius of influence of a single GHCN station measuring precipitation is then described as 30–50 km depending on topology (33). Therefore, tracts 7–30 km from the nearest GHCN station were then considered as a stratum of locations with the next highest expected agreement, and tracts >30 km from the nearest GHCN station were expected to have the lowest agreement.

In order to attempt to isolate social and racial associations with measurement accuracy from other sociogeographic factors potentially related to weather station siting like rurality and terrain, matched subset kappa analyses were performed. Population density was considered as a proxy for rurality and areal topographic prominence was considered as a proxy for terrain. Population density was measured as 1,000 persons/km^2^ using ACS data. United States Geological Survey LANDFIRE elevation data (50) were downloaded at 1 arc-second resolution, then aggregated to 500 m resolution to ease computational requirements, and topographic prominence was calculated as the difference between the highest and lowest elevation in each census tract. Matched analyses compared the agreement of tracts (1) with a social or racial characteristic of interest, (2) without the social or racial characteristic but with population density and topographic prominence within one standard deviation of the referent group, and (3) without the social or racial characteristic and with unmatched population density and topographic prominence.

Population characteristics were compared with respect to each location's average daily distance from the nearest GHCN station using one-way analyses of variance (ANOVA). Comparisons of kappa statistics across strata were made using 95% confidence intervals. Evaluations of sociogeographic determinants of a tracts' distance to the nearest GHCN station were made using linear regression models. Social vulnerability factors found to be associated with distance to nearest GHCN stations through ANOVA were regressed univariably on distance to nearest GHCN station, and multivariably with population density and topographic prominence. All statistical analyses were conducted using R version 4.0.3 and all statistical comparisons were made at the *α* = 0.05 level.

### Local examples

To aid in practical interpretation of these results, locations for three motivating examples were selected. With the strongest observed and predicted increases in EPE frequency in the U.S. occurring in the Northeast (51), and given the particular vulnerability rural residents experience to EPEs and flooding (52), Jefferson County, Pennsylvania was selected to represent a typical rural, Northeast study location. Jefferson County, PA is made up of 13 census tracts in west-central Pennsylvania and in 2020 had a population of 43,846, was 97% non-Hispanic White, 15% of its population were below the federal poverty threshold, and tracts in Jefferson County were on average 14.3 km from the nearest GHCN station. Given the known racial vulnerabilities to the effects of EPEs and climate change (53, 54), the Yakama Indian Reservation in Washington was chosen to represent a typical Native American study location. The Yakama Reservation is located in south-central Washington on the eastern side of the Cascade Mountains and consists of ten census tracts. In 2020, the population in the Yakama Reservation was 54,024, was 45% non-Hispanic White, 13% Native American, 38% Hispanic, 18% of its population were below the federal poverty threshold, and tracts in Yakama Reservation were on average 22.7 km from the nearest GHCN station. Finally, given its ubiquity in early discussions of the effects of climate change through heavy rains and extreme storms (55–58), the New Orleans, Louisiana metropolitan statistical area was selected to represent studies of large urban areas currently experiencing threatening changes to weather and flooding patterns. The New Orleans metropolitan statistical area is made up of eight parishes and 422 census tracts in southern Louisiana centered around the city of New Orleans. In 2020, the area had a population of 1,267,777, was 51% non-Hispanic White, 35% Black, 9% Hispanic, 17% of its population were below the federal poverty threshold, and tracts in the New Orleans metropolitan statistical area were on average 6.2 km from the nearest GHCN station.

For the purpose of illustration, identification of EPEs was considered using the nearest GHCN station data as the reference dataset. We emphasize that this choice of reference does not mean that GHCN is necessarily “correct” for all locations, particularly in areas with low station density. It simply offers a consistent standard against which true and false identification of EPEs in the other datasets can be defined, where “true” and “false” should be interpreted as indicators of agreement or disagreement between datasets. Under this framework, the total count of false positive and false negative EPEs were summed for the tracts in each area from 2011 to 2020 and divided by the total number of tracts to allow for direct comparison in order to illustrate the practical differences among the three data sources and methods for identifying EPEs in different example areas with different demographic profiles and risks of severe impacts of EPEs.

## Results

**Figure 2:**
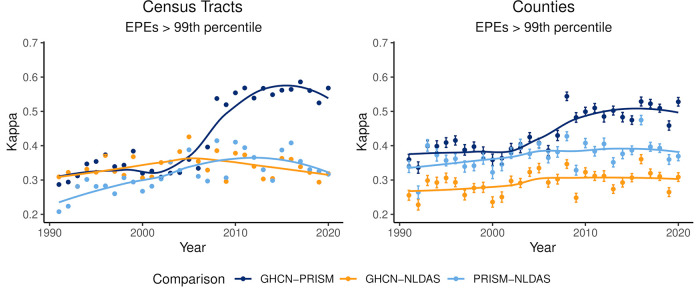
Annual trends in EPE identification agreement according to data source and exposure assessment geography. Point estimates and their 95% confidence intervals for annual Cohen's kappa statistic for comparisons of daily EPE identification for each 2-way comparison between data sources—GHCN vs. PRISM, GHCN vs. NLDAS, and PRISM vs. NLDAS—are presented. A LOESS-smoothed trendline is included to aid in visually identifying temporal trends in agreement. Comparisons among data sources at census tracts appear on the left and comparisons among counties appear on the right.

Over the entire 2020 U.S. Climate Normal period (1991–2020), agreement in identification of EPEs—where daily precipitation exceeded both locally relative and absolute thresholds—was fair to moderate when comparing data from GHCN, PRISM, and NLDAS at the census tract and county levels (Table 1). Overall agreement in identification of EPEs was highest when comparing GHCN and PRISM data and similar when comparing GHCN and NLDAS or PRISM and NLDAS. Identification of EPEs > one inch of precipitation produced higher agreement than EPEs > 99th percentile, and agreement was similar when comparing at the census tract or county level. Agreement was generally higher when identifying EPEs in counties than in census tracts. While differences in the degree of agreement exist depending on the EPE definition, general patterns were consistent between the two definitions and therefore only results from EPEs > 99th percentile are described for the remainder of the analyses. Full results for EPEs > one inch can be found in the Supplementary Material. Agreement between GHCN-NLDAS and between PRISM-NLDAS was relatively stable over time (Figure 2), but agreement in identification of EPEs between GHCN-PRISM data increased substantially around the year 2010, then stabilized in the final decade of the time period of interest. Because of this substantial change in agreement, the following results are presented on analyses performed using precipitation data from 2011 to 2020.

**Table 1:** Agreement in EPE (>99th percentile) identification, 1991–2020.

Geography	GHCN-PRISM	GHCN-NLDAS	PRISM-NLDAS
EPEs > 99th Percentile			
Census Tract	0.446 (0.445–0.446)	0.341 (0.340–0.341)	0.329 (0.328–0.329)
County	0.448 (0.445–0.450)	0.297 (0.294–0.299)	0.378 (0.375–0.380)
EPEs > 1 inch			
Census Tract	0.550 (0.550–0.550)	0.455 (0.454–0.455)	0.439 (0.439–0.439)
County	0.581 (0.580–0.582)	0.422 (0.421–0.423)	0.487 (0.486–0.488)

A number of population characteristics were explored at the census tract level to evaluate their relationship with agreement in the identification of EPEs among the three data sources (Table 2). No significant difference in the overall patterns of agreement among the data sources was noted. When evaluated across the entire CONUS, racial/ethnic differences in population composition were generally not associated with considerable differences in EPE identification agreement. A slight deviation from this finding exists with respect to Hispanic population where Hispanic-majority tracts experienced higher agreement between GHCN-PRISM data in identification of EPEs. However, a significant departure from the overall racial/ethnic patterns in agreement was found with respect to majority Native American tracts. These areas experienced substantially lower agreement in EPE identification when comparing all data sources. While there was not a considerable difference in agreement between the data sources with regard to the income level of census tracts, there was a dose-response type relationship between agreement and SVI score with higher vulnerability relating to lower agreement in EPE identification. However, these patterns were only present when comparing GHCN-PRISM data as GHCN-NLDAS and PRISM-NLDAS agreement statistics were relatively uniform, but much lower. Urbanicity was not shown to affect agreement between regardless of data source comparison; however, mean daily distance from census tracts to the nearest GHCN station had a considerable effect on comparisons of agreement involving GHCN data.

**Table 2:** Census tract population effects on agreement of EPE (>99th percentile) identification (2011–2020).

	GHCN-PRISM	GHCN-NLDAS	PRISM-NLDAS
>50% Racial/Ethnic Minority			
Majority Minority	0.539 (0.537–0.541)	0.342 (0.340–0.344)	0.352 (0.350–0.354)
Non-Majority Minority	0.569 (0.568–0.570)	0.329 (0.328–0.330)	0.350 (0.349–0.351)
>50% Black			
Majority Black	0.559 (0.556–0.562)	0.347 (0.344–0.349)	0.350 (0.347–0.353)
Non-Majority Black	0.561 (0.561–0.562)	0.331 (0.330–0.332)	0.350 (0.349–0.351)
>50% Native American			
Majority Nat. Am.	0.309 (0.288–0.330)	0.226 (0.207–0.244)	0.268 (0.245–0.291)
Non-Majority Nat. Am.	0.562 (0.561–0.563)	0.333 (0.332–0.333)	0.350 (0.350–0.351)
>50% Asian			
Majority Asian	0.432 (0.417–0.446)	0.275 (0.260–0.290)	0.280 (0.266–0.295)
Non-Majority Asian	0.562 (0.561–0.563)	0.333 (0.332–0.333)	0.351 (0.350–0.352)
>50% Hispanic			
Majority Hispanic	0.488 (0.484–0.492)	0.351 (0.348–0.355)	0.360 (0.356–0.364)
Non-Majority Hispanic	0.566 (0.565–0.567)	0.331 (0.330–0.332)	0.350 (0.349–0.351)
Income Level			
Low Income	0.531 (0.523–0.539)	0.352 (0.343–0.360)	0.357 (0.348–0.365)
Non-Low Income	0.562 (0.561–0.562)	0.332 (0.331–0.333)	0.350 (0.349–0.351)
Social Vulnerability Index			
Very Low	0.600 (0.598–0.602)	0.331 (0.329–0.333)	0.351 (0.349–0.353)
Low	0.563 (0.562–0.565)	0.328 (0.326–0.330)	0.348 (0.346–0.349)
High	0.548 (0.546–0.550)	0.329 (0.327–0.331)	0.348 (0.346–0.350)
Very High	0.531 (0.529–0.532)	0.342 (0.340–0.344)	0.355 (0.353–0.357)
Urbanicity			
Rural	0.518 (0.515–0.521)	0.300 (0.298–0.303)	0.347 (0.344–0.350)
Urban Cluster	0.538 (0.536–0.541)	0.322 (0.320–0.324)	0.356 (0.353–0.358)
Urban	0.571 (0.570–0.572)	0.339 (0.338–0.341)	0.350 (0.349–0.351)
Distance to Nearest GHCN Station			
0–7 km	0.594 (0.593–0.596)	0.346 (0.345–0.347)	0.354 (0.353–0.356)
7–30 km	0.518 (0.516–0.519)	0.316 (0.314–0.317)	0.345 (0.344–0.347)
>30 km	0.346 (0.338–0.355)	0.242 (0.235–0.250)	0.310 (0.301–0.319)

Kappa agreement statistics and 95% confidence intervals are presented for each two-way comparison of datasets. Census values were taken from the average of the 2011–2015 and the 2016–2020 5-year American Community Survey estimates. Low income defined as tracts with ≥ 20% population below the federal poverty threshold.

The contribution of sociogeographic characteristics to the observed disparities related to Native American race were explored through a matched sub-analysis. Population density, serving as a measure of rurality, and topographic prominence (measured as difference between highest and lowest elevation), serving as a measure of terrain characteristics, were calculated for each CONUS tract. Kappa statistics were calculated and compared among (1) majority Native American tracts; (2) matched non-majority Native American tracts within one standard deviation of the mean population density and topographic prominence of majority Native American tracts; and (3) unmatched, non-majority Native American tracts (Table 3). After controlling for the potential contribution of rurality and terrain to agreement in EPE exposure assessment, agreement remains only fair in Native American census tracts while it is higher (though still only fair or moderate) in matched and unmatched non-majority Native American tracts.

**Table 3:** Sociogeographically-Matched analysis of native American tracts.

	Native American Tracts	Matched Non-Native American Tracts	Unmatched Non-Native American Tracts
GHCN-PRISM	0.309 (0.288–0.330)	0.535 (0.533–0.537)	0.568 (0.567–0.569)
GHCN-NLDAS	0.226 (0.207–0.244)	0.311 (0.309–0.313)	0.338 (0.337–0.339)
PRISM-NLDAS	0.268 (0.245–0.291)	0.352 (0.350–0.355)	0.350 (0.349–0.351)

Kappa agreement statistics and 95% confidence intervals are presented for each two-way comparison of datasets among three strata of U.S. census tracts: (1) majority Native American tracts, (2) matched non-majority Native American tracts within one standard deviation of the mean population density and topographic prominence of majority Native American tracts, and (3) unmatched, non-majority Native American tracts.

**Figure 3:**
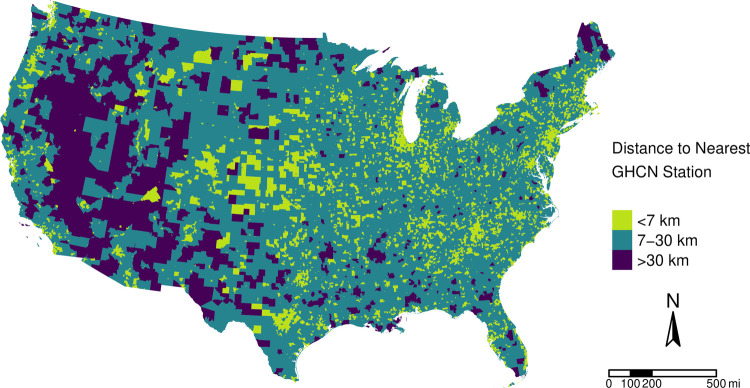
Census tract distance to nearest GHCN station, 2011–2020. Mean daily distances from census tract centroid to the nearest GHCN station from 2011 to 2020 are presented. Distances are binned according to their reliability at representing precipitation observed at that location. Precipitation values estimated for census tracts >30 km from the nearest GHCN station are less reliable as they are located at or beyond the limit of the radius of influence of a GHCN station (30–50 km depending on topology).

Given the dependency of agreement in EPE identification on distance from the nearest GHCN station, geographic and population characteristics of these census tracts are presented in Figure 3 and Table 4. 42,971 (59%) tracts are located within 7 km of a GHCN station, 28,465 (39%) are 7–30 km away, and 897 (1%) tracts are further than 30 km from the nearest GHCN station. Tracts located >30 km from the nearest GHCN station can be found throughout the entire CONUS but are generally concentrated in the Great Basin region of Oregon, Nevada, and California and in the Canyon Lands region of Utah and Arizona. Residents of tracts located >30 km from the nearest GHCN station tend to be non-majority minority areas, but have 11.2 times more Native American residents, 40% more residents below the federal poverty threshold, and have an average SVI 15 percentile points higher than tracts within 30 km of the nearest GHCN station. While tracts >30 km from the nearest GHCN station represent a very small proportion of the total number of tracts and overall population, there are over 3 million individuals for whom the nearest precipitation monitor is located at or beyond the radius of influence of a single station.

**Table 4:** Census tract population characteristics over GHCN distance Strata.

	Distance to GHCN Station			One-way ANOVA *p*
	<7 km	7–30 km	>30 km	
Total Population*	189,851,002 (60%)	123,800,885 (39%)	3,015,042 (1%)	—
Minority Population (%)	41.6 (41.5–41.7)	32.6 (32.5–32.7)	35.1 (34.4–35.8)	<0.0001
Black Population (%)	15.3 (15.2–15.4)	11.8 (11.7–11.9)	9.6 (9.2–10.1)	<0.0001
Native American Population (%)	0.6 (0.6–0.6)	1.0 (1.0–1.0)	8.5 (8.0–9.1)	<0.0001
Asian Population (%)	5.7 (5.7–5.7)	3.4 (3.4–3.4)	0.7 (0.6–0.7)	<0.0001
Hispanic Population (%)	17.7 (17.6–17.8)	14.4 (14.3–14.5)	14.6 (14.1–15.1)	<0.0001
Poverty (%)	15.1 (15.0–15.1)	14.8 (14.7–14.8)	19.4 (19.2–19.7)	<0.0001
Social Vulnerability Index	0.49 (0.49–0.49)	0.51 (0.51–0.51)	0.65 (0.65–0.66)	<0.0001
Median Income ($)	33,700 (33,651–33,750)	30,220 (30,172–30,267)	24,024 (23,877–24,172)	<0.0001

Mean values and 95% confidence intervals are presented for census data taken from the average of the 2011–2015 and the 2016–2020 5-year American Community Survey estimates are presented for strata of census tracts whose average daily distance to the nearest GHCN station is <7 km, 7–30 km, or >30 km.

* Total population and percent of total CONUS population.

Sociogeographic contributions to the disparity in GHCN station proximity to Native American-majority tracts were also evaluated through linear regression models (Table 5). Increasing levels of Native American population (%) were associated with further distance from the nearest GHCN station. This significant association remained after controlling for topographic prominence and population density, though topographic prominence substantially dampened the effect, suggesting that terrain in Native American-majority tracts may impact the siting of GHCN stations.

**Table 5:** Associations between census tract sociogeographic characteristics and distance to nearest GHCN station.

	Model 1	Model 2	Model 3	Model 4
Intercept	7.44 (7.40–7.49)	6.89 (6.85–6.93)	7.85 (7.80–7.90)	7.21 (7.16–7.26)
% Native American	26.94 (25.93–27.94)	19.50 (18.54–20.46)	26.28 (25.28–27.27)	19.32 (18.36–20.27)
Topographic Prominence*	—	1.05 (1.03–1.08)	—	1.01 (0.99–1.03)
Population Density^†^	—	—	−0.20 (−0.21 to −0.19)	−0.14 (−0.15 to −0.13)

Linear regression model coefficients and 95% confidence intervals of sociogeographic tract variables regressed on average daily distance (in km) to nearest GHCN station are presented. Positive regression coefficients represent a direct association with increased distance from the nearest GHCN station.

* Tract topographic prominence measured as difference between highest and lowest elevation in km.

**^†^**Population density calculated as 1,000 population/km^2^.

### Local examples

Compared to the overall kappa statistic for the entire CONUS from 2011 to 2020, patterns of agreement vary widely depending on the location and data source used (Table 6). In more practical terms, this range of agreement, which varies from moderate in some areas to only slight in others, results in substantial differences in EPE identification. Under this framework, PRISM and NLDAS do not show an EPE in over half of the cases in each of the local example areas when the nearest GHCN station does (Table 7; 2011–2020 data). In the Yakama Reservation, WA, these results are particularly striking—PRISM and NLDAS data identify only five of the same GHCN-defined EPEs > the 99th percentile. Interpretation of these results must be treated carefully, however. While GHCN data are referred to as “truth” for the purposes of this example, no claim is made that these data are actually the most accurate when comparing at grid scale, nor that precipitation values estimated for sites away from the station location using GHCN data reflect the true experienced values. Though it is quite likely that PRISM or NLDAS offer more accurate diagnosis of EPEs for remote sites far from a weather station, GHCN is simply used here as the reference dataset for comparisons.

**Table 6:** Local examples of EPE identification (> 99th percentile), 2011–2020.

	GHCN-PRISM	GHCN-NLDAS	PRISM-NLDAS
Overall U.S.	0.560 (0.560–0.561)	0.332 (0.331–0.333)	0.350 (0.349–0.351)
Jefferson County, PA	0.293 (0.230–0.355)	0.448 (0.382–0.513)	0.200 (0.143–0.255)
Yakama Reservation, WA	0.158 (0.120–0.195)	0.145 (0.109–0.181)	0.375 (0.291–0.459)
New Orleans, LA	0.474 (0.463–0.485)	0.423 (0.420–0.426)	0.383 (0.379–0.386)

Kappa agreement statistics and 95% confidence intervals are presented for each two-way comparison of datasets at three example locations. Overall kappa statistic for agreement across the entire conterminous U.S. is presented for comparison to full results.

**Table 7:** Local examples of EPE false positives and false negatives – PRISM vs. GHCN, 2011–2020.

		PRISM		NLDAS	
	GHCN EPEs*	False Positives	False Negatives	False Positives	False Negatives
Jefferson Co., PA	13	10	9	8	7
Yakama Res., WA	57	3	52	7	52
New Orleans, LA	16	6	9	7	11

* EPEs identified by GHCN were considered as “truth” for the purposes of this example, though no claim is made that these data are actually the most accurate when comparing at grid scale, nor that precipitation values estimated for sites away from the station location using GHCN data reflect the true experienced values. The terms “false positives” and “false negatives,” therefore, do not provide a description of true measurement accuracy, but rather describe how often each dataset agrees in its identification of an EPE on a given day.

## Discussion

The objectives of this study were to characterize the agreement among three commonly used meteorological data sources and methods in their identification of EPEs in the CONUS over the 1991–2020 U.S. Climate Normals period and to evaluate the association between sociodemographic population characteristics with agreement in EPE identification. Overall agreement in EPE identification among the nearest GHCN station, spatially weighted average PRISM value, and spatially weighted average NLDAS values was fair to moderate. We identified the potential for these three commonly used classes of data sources for EPE classification to vary according to vulnerability status. When comparing GHCN and PRISM data, there was a substantially lower level of agreement in census tracts with > 50% Native American population, and increasing SVI scores were associated with lower agreement in EPE identification, suggesting the possibility for differential exposure misclassification according to community vulnerability to the harmful effects of EPEs.

The severity of impacts of climate change on health exist on a continuum, with some populations being more protected and others being more at risk. Some of these populations are referred to as susceptible, meaning they are biologically predisposed to more severe health outcomes, while others are considered vulnerable, meaning there are societal and structural issues that place them at higher risk (59). While investigations in the heat and health literature identify ways that data source decisions can affect measures of association by changing exposure misclassification rates, results from the present study show that while different data sources may decrease levels of measurement error and minimize the impact of exposure misclassification, these changes do not occur uniformly across strata of many common environmental and community determinants of health such as race, socioeconomic status, and social vulnerability, which vary substantially over space (49, 60–62). These factors all increase a population's vulnerability to negative health effects of climate change (53, 54, 60, 63–67). The results presented here demonstrate that the evaluated data sources and methods vary across commonly identified vulnerability factors, suggesting that different exposure data may limit the ability to detect harmful health effects of climate change in a population at higher risk. As investigations of environmental and climate justice issues continue to emerge as an important area of concern for researchers and funding organizations, the concerns identified in the present study highlight an important potential limitation of researchers to conduct these investigations using the existing climate and meteorological infrastructure available in wealthy, developed countries like the United States (these issues are far more prevalent in low- and middle-income countries) (68). The disappointing irony of this situation is that researchers may not be able to accurately assess exposures to climate hazards in the very communities that are most vulnerable to the effects of those hazards, potentially leading to an inability to accurately characterize associations between weather exposures and health outcomes that could identify areas most in need of interventions, resources, or further study.

Native Americans specifically were identified as a vulnerable population for whom challenges in accurate exposure assessment were found. These challenges are primarily related to the proximity of Native American-majority areas to first-order weather monitoring stations, a factor that was found to be affected by the terrain of these areas; however, the Native American composition of census tracts nonetheless remained significantly associated with proximity to the nearest GHCN station even after adjustment for terrain and population density. This is an especially prescient finding given the unique threats that climate change poses to Indigenous physical and mental health, economies, and livelihoods (54), challenges that are not accidental. Farrell (69) found that Indigenous lands in the CONUS have been reduced by nearly 99% in area, and that the lands to which Native peoples have been displaced expose residents to greater average climate hazards. In fact, the Quinault Indian Nation, located on the Pacific coast on the Olympic Peninsula in Washington, plans to relocate in response to rising sea levels and increased threats of tsunamis, storm surge, and river flooding (70, 71). However, many federal Indigenous areas are located in arid climates with small predicted risks of EPEs throughout the coming century (51), areas where the importance of accurate assessment of exposures to EPEs might not seem to be as important, though the various other effects brought about by rising global temperatures that might affect these communities—drought, extreme heat, and wildfire risk (28, 37, 69)—all benefit from accurate exposure assessment characterization as well. Beyond findings related to specific vulnerable groups, a more general measure of social vulnerability, the Centers for Disease Control and Prevention Agency for Toxic Substances and Disease Registry's SVI was also correlated with moderate to poor agreement in exposure assessment among the three evaluated data sources. SVI scores have been associated with climate and environmental disaster-related health outcomes like heat-related illness and mortality, wildfires and vulnerability to climate extremes, and tropical storms (49, 72–76) further highlighting the importance of accurate assessment of exposures to climate extremes in these populations at increased risk.

In the United States, the Interagency Working Group on Climate Change and Health explored existing research on the health implications of weather-related morbidity and mortality and identified several key research needs, among them better characterizations of the health impacts of extreme weather events, improvements to the predictive power of modeling the health effects of extreme events, and development of regional climate models for early warning of extreme events (67). While existing weather monitoring infrastructure provides excellent spatial and population coverage of the CONUS (99% of the population resides within the 30 km radius of influence of a precipitation monitoring station), the vulnerable sociodemographic makeup of the remaining 1%—more than three million individuals for whom exposure to intense rain and snowfall, drought, and extreme heat or cold is not well-characterized—highlights the need for further investment in reliable meteorological monitoring systems that will enhance not only academic study of population effects of climate change, but also increase the accuracy of early warning and real-time meteorological modeling and forecasting for these vulnerable populations. While the present investigation found that proximity to the nearest ground station was a strong indicator of agreement among exposure datasets, there was still not perfect agreement even in areas very close to first-order stations, suggesting substantial differences among the datasets likely driven by modeling components. These include elevation and orography for PRISM-modeled data; complex land-surface modeling involving temperature, humidity, surface pressure, wind, and other factors for NLDAS-modeled data; and a number of other factors specific to the many alternative gridded precipitation modeling datasets that were not explored here. These distinctions necessitate a nuanced approach to the selection of the dataset chosen to represent exposure assessment for a given study population that understands the sources of error and bias for observed and interpolated model components. Still, the use of gridded datasets clearly improves the spatial resolution of weather monitoring for extreme events, a definite benefit for vulnerable populations located far from existing weather monitoring infrastructure. However, the gridded datasets evaluated here and indeed many alternative gridded climate datasets rely on or incorporate first-order weather station observations, so improvements to the spatial distribution of this network of monitoring sites would have downstream benefits to the accuracy and precision of nearly any climate dataset. Nevertheless, while improvement of measurement accuracy through increased station density may serve to provide meteorologists, climatologists, and epidemiologists with better tools to measure and study community-level effects of climate change and extreme weather, it is imperative that such improvements meet community-identified needs. Indeed, the most important first step to addressing racial inequity in climate-based threats to health is to work with affected and vulnerable communities to define what is measured, what is not measured but needs to be measured, and how to gauge success or failure (77).

PRISM precipitation data previously have been validated against ground station observations, and they have generally been found to have good agreement with precipitation measurements made at ground stations with low absolute error and minimal regional biases (23, 78–80). NLDAS precipitation data have also been compared against ground station observations and while high temporal-scale (i.e., hourly) measurements experienced large deviations, daily and longer-term observation periods were highly similar (81). However, these validation studies were only able to compare values at known locations and while observations at ground stations are considered the “gold standard” for meteorological conditions experienced at that location, population distributions are often dispersed far from these stations, leading to misclassification when these values are used as a proxy of individual or population exposure (82). It is important to note, however, that there was also no “gold standard” for the purpose of comparison or validation in this study. Good vs. poor agreement in the results reported here, therefore, is not a reflection of model performance, but rather an indicator that model selection may have a significant effect on subsequent analyses depending on the strengths and limitations of a given dataset's specific application. Beyond the sociodemographic and temporal factors demonstrated to be related to exposure assessment agreement here, there are also geographic factors that deserve consideration. Understanding these limitations, the results of these analyses cannot support the recommendation of any specific data source or modeling method, but rather reinforce the importance of careful consideration of the strengths and limitations of the full range of climatological datasets available to represent the exposures of a given population. For health applications, the actual “gold standard” is personal exposure assessment (83). However, epidemiologic research involving personal climate exposure is often financially or logistically unfeasible, and therefore high-spatiotemporal resolution gridded climate datasets are often a reasonable proxy for individual exposure to ambient meteorological conditions. In truth, estimates of meteorological conditions from fine-scale interpolated or downscaled systems may ultimately decrease the measurement error from systematic differences observed between ground station observations and the true conditions a study population experiences (24). The use of a single observation to represent multiple individuals is also subject to Berkson measurement error which, while not expected to bias effect estimates, can lead to decreased precision in effect estimates and smaller effect sizes (84, 85). Finally, agreement between PRISM estimates and GHCN observations was seen in this analysis to increase substantially in the late 2000s. The two most likely explanations are a change to the PRISM modeling methods in 2008 (33) and a large increase in the number of GHCN stations that came online around the same time (86), both of which would serve to increase levels of agreement.

Differences in exposure assessment, biases, measurement errors, and exposure misclassification can have substantial impacts on effect estimates, effect sizes, and internal validity when correlating climate exposures with health outcomes. While we are not aware of similar comparisons of first-order stations with gridded climate data on precipitation or EPEs, investigations into the practical implications of dataset choice have been conducted with respect to temperature, heat index, and extreme heat. In an investigation of 113 U.S counties from 1987 to 2006, Weinberger et al. (24) compared the risk of heat-related mortality using temperature data from single stations using the Integrated Surface Database Lite with temperature data using PRISM. Due to the lack of spatial resolution when assigning a single station's observation as the temperature experienced by an entire county, the authors found that risk ratios of heat-related mortality were higher using the enhanced spatial resolution data from PRISM compared to the single stations. Clemens et al. (20) investigated heat- and heat index-associated mortality in Ontario, Canada from 2005 to 2012 using weather station data and 1 km × 1 km gridded temperature data and found that the added spatial variability afforded by the gridded data sources provided higher estimates of daily maximum temperature and narrower confidence intervals in calculations of the daily relative risk of mortality. In a similar analysis of heat-related mortality in Switzerland and the United Kingdom, de Schrijver et al. (21) explored the effect of data from weather stations and three gridded climate datasets at 1.6 km × 2.3 km, 5 km × 5 km, and 0.25° × 0.25° spatial resolution. While they did not find substantial differences in the measures of association or effect sizes, use of the high-resolution data products provided better predictive ability of statistical models. Practically speaking, when planning a study exploring the effects of EPEs, spatial resolution of the exposure dataset and the areal unit to which exposures are being assigned play into the accuracy of exposure measurements, but so too does the definition used to identify EPEs. In this analysis, locally specific 99th percentile thresholds demonstrated lower agreement than an absolute threshold of one inch. While alternative definitions exist that were not explored here, these findings suggest that more strict definitions resulting in rarer event occurrences might be more susceptible to the influence of measurement errors and biases among potential datasets.

These findings represent practical analyses of commonly used methods in the published climate literature. The data sources selected are widely used within the established literature and the more nascent field of the health effects of precipitation and climate, and evaluations were made at spatial units to which epidemiologic data are commonly aggregated. Extreme events are observed and predicted with high confidence to increase with increasing global temperatures and their frequency is reliably attributed to anthropogenic causes (28, 37). The calculation of the kappa agreement statistic is sound and robust to the effects of random agreement when outcome events are rare (45). However, this study does have a number of limitations. First, only two gridded datasets were used among the many that exist, and the simplest methods for considering ground station data were used. Alternative uses that might be considered in future work could incorporate multiple ground stations through spatial interpolation modeling like inverse distance weighting or kriging for comparison against gridded climate datasets. Second, while many different valid definitions for EPEs exist, only two were considered here. Though these two definitions are commonly used in climate and health literature, alternative definitions of EPEs may have resulted in different relative performance of the climate datasets. Relatedly, comparisons were made using extreme event occurrences, not actual precipitation values. While many health effects and climate predictions involve extreme events, the performance of different sources of daily precipitation measurements may have some bearing on research in other fields, such as infectious, allergic or respiratory disease outcomes, air quality exposures, agricultural productivity, or planetary health factors and should be considered by future research. While important modifiable community vulnerability factors were considered, there are likely differences in the agreement and performance of these and other climate datasets according to seasonality, geography, elevation, and orography that were not explored here. While the data for these analyses were taken from the entire 1991–2020 U.S. Climate Normals time period, the associations with race and social and vulnerability were detected using a 10-year subset (2011–2020) due to systematic changes in the GHCN and PRISM methods that would have complicated interpretation of analyses involving the full 30-year period. Geographic centroids and area-weighted averages were used to approximate population exposure using each of the three datasets rather than calculating population centroids and population-weighted areal averages. Finally, more complex statistical modeling involving the use of mixed methods were considered to allow for the evaluation of agreement and correlation across continuous scales of modifying factors, but due to the very large size of the datasets under consideration, this approach was found to be computationally unfeasible. However, future work may be able to apply such an approach to a smaller regional analysis or over a shorter timeframe, and may also include sociogeographic and other variables to develop a better understanding of the factors related to accurate exposure assessment of extreme climate events.

## Conclusion

Agreement in identification of EPEs among GHCN, PRISM, and NLDAS is fair to moderate from 1991 to 2020. There are spatial and temporal differences in the levels of agreement between ground stations and gridded climate datasets in their detection of EPEs in the U.S. Spatial variation in agreement is most strongly related to the proximity to the nearest ground station, with areas furthest from a ground station demonstrating the lowest levels of agreement. These areas include areas that are lower socioeconomic status, have a higher Native American population, and have higher SVI scores. The addition of ground stations in these areas may increase agreement, and future studies hoping to use these or similar data sources should be aware of the limitations, biases, and potential for differential exposure misclassification across a potential study population. Most importantly, vulnerable populations should be engaged to determine their priorities for enhanced surveillance of climate-based threats so that community-identified needs are met by any future improvements in data quality.

## Data Availability

Publicly available datasets were analyzed in this study. These data can be found here: https://www.ncei.noaa.gov/products/Land-based-station/global-historical-climatology-network-daily; https://www.prism.oregonstate.edu/; https://disc.gsfc.nasa.gov/.
