# Supplementary material for: Agreement in extreme precipitation exposure assessment is modified by race and social vulnerability
Source: Front Epidemiol. 2023 Mar 2;3:1128501. doi: 10.3389/fepid.2023.1128501 (PMC10911001; doi:10.3389/fepid.2023.1128501)
Supplement: Supplementary file 1 [file Datasheet1.docx]

Supplementary Material

Agreement in Extreme Precipitation Exposure Assessment is Modified by Race and Social Vulnerability

# Supplementary Figures and Tables

## Supplementary Figures

Supplementary Figure 1. Annual Trends in EPE (> 1 Inch) Identification Agreement According to Data Source and Exposure Assessment Geography


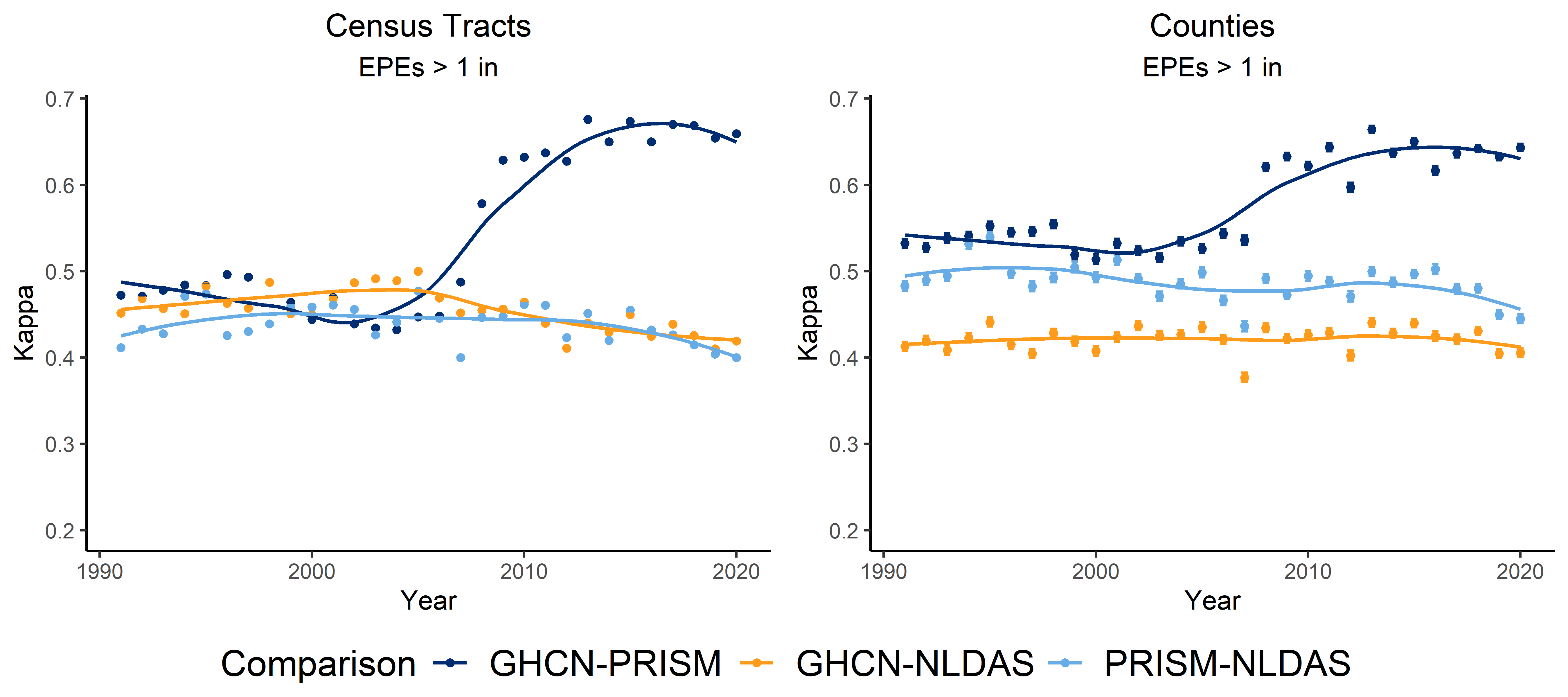


**Supplementary Figure 1.** Point estimates and their 95% confidence intervals for annual Cohen’s kappa statistic for comparisons of daily EPE (defined as days exceeding one inch**)** identification for each 2-way comparison between data sources – GHCN vs. PRISM, GHCN vs. NLDAS, and PRISM vs. NLDAS – are presented. A LOESS-smoothed trendline is included to aid in visually identifying temporal trends in agreement. Comparisons among data sources at census tracts appear **o**n the left and comparisons among at counties appear **o**n the right**.**

## Supplementary Tables

Supplementary Table 1. Census Tract Population Effects on Agreement of EPE (> 1 Inch) Identification (2011-2020)

|  |  | GHCN-PRISM | GHCN-NLDAS | PRISM-NLDAS |
| --- | --- | --- | --- | --- |
| >50% Minority Population | |  |  |  |
|  | Majority Minority | 0.61 (0.613-0.614) | 0.44 (0.437-0.438) | 0.42 (0.420-0.421) |
|  | Non-Majority Minority | 0.68 (0.677-0.678) | 0.43 (0.425-0.426) | 0.43 (0.432-0.433) |
| >50% Black Population | |  |  |  |
|  | Majority Black | 0.62 (0.615-0.617) | 0.43 (0.429-0.431) | 0.42 (0.417-0.419) |
|  | Non-Majority Black | 0.66 (0.664-0.664) | 0.43 (0.429-0.429) | 0.43 (0.430-0.430) |
| >50% Native American Population | |  |  |  |
|  | Majority Native American | 0.51 (0.495-0.517) | 0.40 (0.390-0.414) | 0.44 (0.429-0.454) |
|  | Non-Majority Native American | 0.66 (0.658-0.659) | 0.43 (0.429-0.430) | 0.43 (0.428-0.429) |
| >50% Hispanic Population | |  |  |  |
|  | Majority Hispanic | 0.57 (0.564-0.567) | 0.46 (0.461-0.463) | 0.43 (0.424-0.427) |
|  | Non-Majority Hispanic | 0.66 (0.665-0.665) | 0.43 (0.427-0.427) | 0.43 (0.428-0.429) |
| Income Level | |  |  |  |
|  | Low Income | 0.60 (0.598-0.603) | 0.44 (0.434-0.440) | 0.42 (0.416-0.422) |
|  | Non-Low Income | 0.66 (0.659-0.660) | 0.43 (0.429-0.430) | 0.43 (0.428-0.429) |
| Social Vulnerability Index | |  |  |  |
|  | Very Low | 0.70 (0.698-0.700) | 0.42 (0.421-0.423) | 0.42 (0.423-0.425) |
|  | Low | 0.67 (0.670-0.671) | 0.43 (0.425-0.427) | 0.43 (0.429-0.430) |
|  | High | 0.65 (0.651-0.652) | 0.43 (0.429-0.430) | 0.43 (0.432-0.433) |
|  | Very High | 0.61 (0.614-0.616) | 0.44 (0.439-0.441) | 0.43 (0.428-0.430) |
| Urbanicity | |  |  |  |
|  | Rural | 0.66 (0.663-0.665) | 0.42 (0.418-0.421) | 0.45 (0.453-0.455) |
|  | Urban Cluster | 0.67 (0.673-0.674) | 0.43 (0.431-0.433) | 0.45 (0.454-0.455) |
|  | Urban | 0.65 (0.653-0.654) | 0.43 (0.430-0.430) | 0.42 (0.419-0.420) |
| Distance to Nearest GHCN Station | |  |  |  |
|  | 0-7km | 0.68 (0.678-0.678) | 0.44 (0.439-0.440) | 0.43 (0.429-0.430) |
|  | 7-30km | 0.63 (0.632-0.632) | 0.42 (0.415-0.417) | 0.43 (0.426-0.427) |
|  | >30km | 0.54 (0.532-0.539) | 0.38 (0.378-0.385) | 0.44 (0.439-0.446) |

Supplementary Table 1. Kappa agreement statistics presented for each two-way comparison of datasets for EPEs defined as days with precipitation > one inch. Census values taken from 2016-2020 5-year American Community Survey estimates. Low income defined as tracts with ≥ 20% population below the poverty limit.

Supplementary Table 2. Local Examples of EPE Identification (> 1 Inch), 2011-2020

|  | GHCN-PRISM | GHCN-NLDAS | PRISM-NLDAS |
| --- | --- | --- | --- |
| Overall U.S. | 0.56 | 0.33 | 0.35 |
| Jefferson County, PA | 0.37 | 0.41 | 0.32 |
| Yakama Reservation, WA | 0.51 | 0.46 | 0.52 |
| New Orleans, LA | 0.49 | 0.42 | 0.38 |

Supplementary Table 2. Kappa agreement statistics presented for each two-way comparison of datasets. Overall kappa statistic for agreement across the entire conterminous U.S. is presented for comparison to full results.

Supplementary Table 3. Local Examples of EPE (> 1 Inch) False Positives and False Negatives – PRISM vs. GHCN, 2011-2020

|  |  | PRISM vs GHCN | | NLDAS vs GHCN | |
| --- | --- | --- | --- | --- | --- |
|  | Total EPEs* | False Positives | False Negatives | False Positives | False Negatives |
| Jefferson Co., PA | 82 | 53 | 50 | 24 | 54 |
| Yakama Res., WA | 33 | 12 | 17 | 13 | 19 |
| New Orleans, LA | 224 | 83 | 117 | 72 | 138 |

Supplementary Table 3. * EPEs identified by GHCN
